# Optimal two-stage strategy for detecting interacting genes in complex diseases

**DOI:** 10.1186/1471-2156-7-39

**Published:** 2006-06-15

**Authors:** luliana lonita, Michael Man

**Affiliations:** 1 Courant Institute of Mathematical Sciences, New York University, 251 Mercer Street, New York, NY, 10012, USA; 2 Nonclinical Statistics, Pfizer PGRD, 2800 Plymouth Rd, Ann Arbor, Ml, 48105, USA

## Abstract

**Background:**

The mapping of complex diseases is one of the most important problems in human genetics today. The rapid development of technology for genetic research has led to the discovery of millions of polymorphisms across the human genome, making it possible to conduct genome-wide association studies with hundreds of thousands of markers. Given the large number of markers to be tested in such studies, a two-stage strategy may be a reasonable and powerful approach: in the first stage, a small subset of promising loci is identified using single-locus testing, and, in the second stage, multi-locus methods are used while taking into account the loci selected in the first stage. In this report, we investigate and compare two possible two-stage strategies for genome-wide association studies: a conditional approach and a simultaneous approach.

**Results:**

We investigate the power of both the conditional and the simultaneous approach to detect the disease loci for a range of two-locus disease models in a case-control study design. Our results suggest that, overall, the conditional approach is more robust and more powerful than the simultaneous approach; the conditional approach can greatly outperform the simultaneous approach when one of the two disease loci has weak marginal effect, but interacts strongly with the other, stronger locus (easily detectable using single-locus methods in the first stage).

**Conclusion:**

Genome-wide association studies hold the promise of finding new genes implicated in complex diseases. Two-stage strategies are likely to be employed in these large-scale studies. Therefore we compared two natural two-stage approaches: the conditional approach and the simultaneous approach. Our power studies suggest that, when doing genome-wide association studies, a two-stage conditional approach is likely to be more powerful than a two-stage simultaneous approach.

## Background

The mapping of complex diseases is one of the most important problems in human genetics today. The rapid development of technology has led to the discovery of millions of polymorphisms across the human genome, making it possible to conduct genome-wide association studies with hundreds of thousands of markers.

The single-locus approach to mapping complex traits evaluates the marginal effect at each marker in turn, thereby ignoring potentially useful gene-gene interaction effects. Alternative approaches, that take into account both marginal and interactive effects, may offer increased power, especially when disease genes have only moderate marginal effect, but work together (interact) in the manifestation of a disease [1]. A natural strategy when dealing with a large number of markers is a two-stage method: in the first stage, a single-locus approach is used to select promising markers by employing a liberal significance criterion; in the second stage, multi-locus approaches that capture both marginal and interaction effects are used to discover loci at a stringent significance level.

Several promising two-stage approaches have recently been proposed in the literature [2,3]. In this report, our goal is to investigate and compare two possible two-stage strategies. The first one was used in the paper by Marchini et al. [2]. That method is based on first selecting loci at a liberal significance level and subsequently testing all the pairwise combinations of the loci selected in the first stage. We will refer to this as the simultaneous method. The second strategy is the so-called conditional approach (as suggested also by Daly and Altschuler [4]) frequently used in genetic studies nowadays. Namely, a single-locus screening is performed in the first stage to select loci at a more stringent level, and in the second stage pairwise combinations between the selected loci and the initial set of loci are being analyzed. We use simulation to address the following two questions: first, what is the optimal cutoff (the significance level) for each method to use in the first stage in order to obtain the highest power to detect both disease loci at a genome-wide error level of at most 0.05, and second, which of the two strategies mentioned above performs better.

## Results and discussion

For power computation we use an analytical approach: the "exemplary data" approach of Longmate[5]. The exemplary data approach provides a general method of estimating the power of likelihood-ratio tests in complex disease models. In this approach a disease model is specified with the conditional penetrance matrix, given each configuration of risk genotypes, and the joint distribution of risk genotypes at the disease loci. Then an exemplary data set is generated that represents the expected data under the sampling design. The exemplary data set is then analyzed, computing any desired likelihood-ratio test. The likelihood-ratio can be used to compute the power of the same test when applied to data sets simulated under the same scenario (for details see [5]).

We simulate case-control data under several epistatic models: two-locus threshold, two-locus multiplicative, and two-locus missing lethal genotype. In the two-locus threshold model, the odds of disease have a baseline value (*α*) unless a disease allele is present at each locus. Once this threshold is reached, the odds of disease increase to *α*(1 + *θ*). The missing lethal genotype disease model is similar to a threshold model, in the sense that a minimum number of disease alleles are required from both loci. However if the disease is lethal, all individuals carrying a large number of disease alleles disappear from the population. And for the multiplicative models, the odds of disease increase multiplicatively both within and between genotypes once there is at least one disease allele at each disease locus. Odds of disease for these models are given in Tables 1, 2, 3.

**Table 1:** Threshold Model. Odds of disease for the threshold disease model.

Model 1			
	bb	Bb	BB

aa	*α*	*α*	*α*
Aa	*α*	*α*(1 + *θ*)	*α*(1 + *θ*)
AA	*α*	*α *(1 + *θ*)	*α*(1 + *θ*)

**Table 2:** Missing Lethal Genotype Model. Odds of disease for the missing lethal genotype disease model.

Model 2			
	bb	Bb	BB

aa	*α*	*α*	2*α*(1 + *θ*)
Aa	*α*	*α*(1 + *θ*)	*α*
AA	2*α*(1 + *θ*)	*α*	*α*

**Table 3:** Multiplicative Model. Odds of disease for the multiplicative disease model.

Model 3			
	bb	Bb	BB

aa	*α*	*α*	*α*
Aa	*α*	*α*(1 + *θ*)	*α*(1 + *θ*)^2^
AA	*α*	*α*(1 + *θ*)^2^	*α*(1 + *θ*)^4^

The minor allele frequency (MAF) at each disease locus is chosen from {0.05, 0.2, 0.5}, *α *= 0.05 and the interaction parameter *θ *∈ {1.5, 2.5}. We simulate *n *= 1000 cases and *n *= 1000 controls and 300,000 markers. Two of the markers are the disease loci. The markers are not linked among themselves (i.e. they are independent genetic loci).

**Figure 1:**
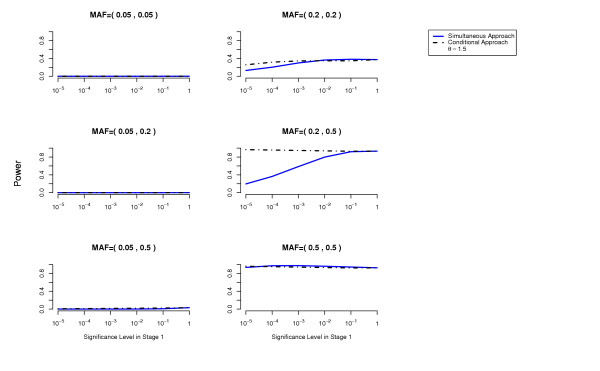
**Threshold Model**. Comparison of the conditional approach with the simultaneous approach for the two-locus threshold disease model. *θ *= 1.5. Each picture corresponds to a specific combination of MAFs at the two disease loci as indicated above each plot.

**Figure 2:**
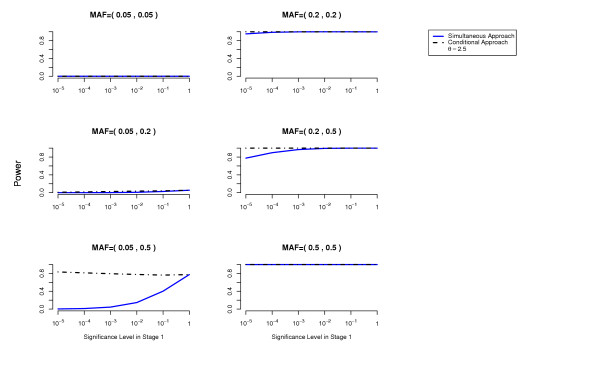
**Threshold Model**. Comparison of the conditional approach with the simultaneous approach for the two-locus threshold disease model. *θ *= 2.5. Each picture corresponds to a specific combination of MAFs at the two disease loci as indicated above each plot.

**Figure 3:**
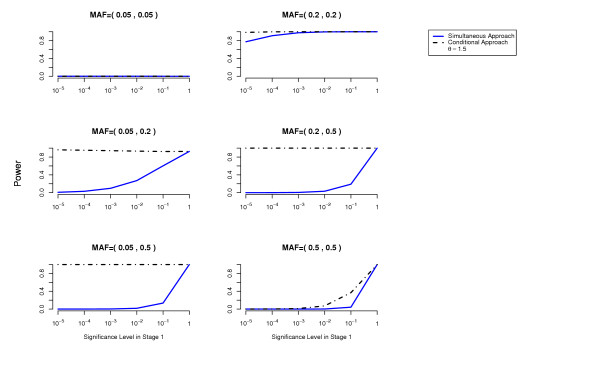
**Missing Lethal Genotype Model**. Comparison of the conditional approach with the simultaneous approach for the two-locus missing lethal genotype disease model. *θ *= 1.5. Each picture corresponds to a specific combination of MAFs at the two disease loci as indicated above each plot.

**Figure 4:**
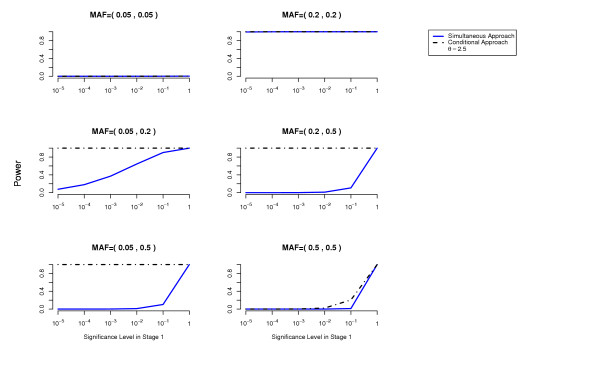
**Missing Lethal Genotype Model**. Comparison of the conditional approach with the simultaneous approach for the two-locus missing lethal genotype disease model. *θ *= 2.5. Each picture corresponds to a specific combination of MAFs at the two disease loci as indicated above each plot.

**Figure 5:**
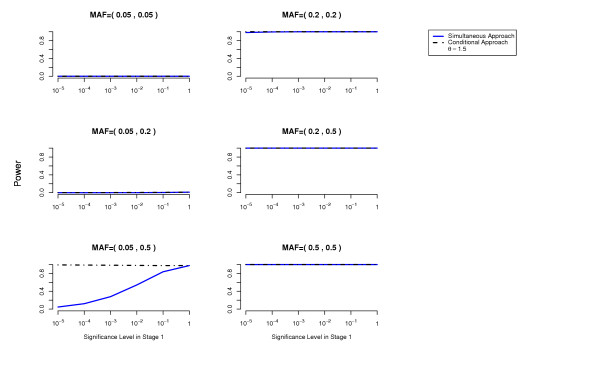
**Multiplicative Model**. Comparison of the conditional approach with the simultaneous approach for the two-locus multiplicative disease model. *θ *= 1.5. Each picture corresponds to a specific combination of MAFs at the two disease loci as indicated above each plot.

**Figure 6:**
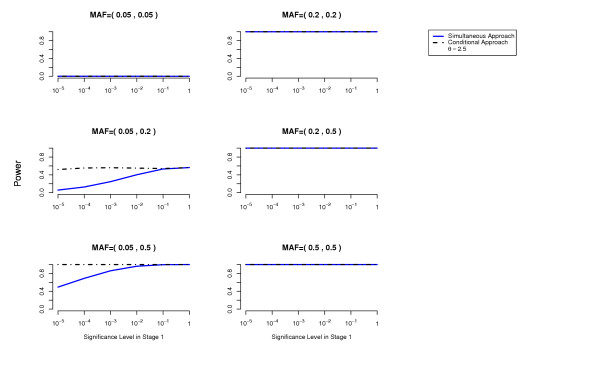
**Multiplicative Model**. Comparison of the conditional approach with the simultaneous approach for the two-locus multiplicative disease model. *θ *= 2.5. Each picture corresponds to a specific combination of MAFs at the two disease loci as indicated above each plot.

We compute the power to detect both disease loci as a function of the cutoff used in the first stage for each of the two methods (simultaneous and conditional) (details about these power computations are given in the Methods Section). The overall Type 1 error is at most 0.05 (using a Bonferroni correction to account for multiple testing). The results are presented in Figures 1, 2, 3, 4, 5, 6.

For the conditional approach, a more stringent cutoff can be used in the first stage (in our simulated examples 10^-5 ^or 10^-4^), whereas for the simultaneous approach a more liberal cutoff (for example 10^-1^) is essential.

**Figure 7:**
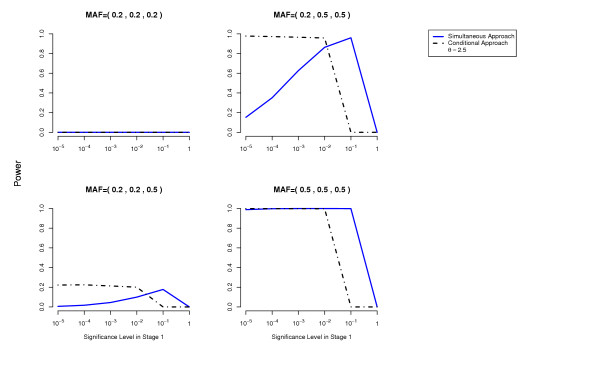
**Three-locus Threshold Model**. Comparison of the conditional approach with the simultaneous approach for the three-locus threshold disease model. *θ *= 2.5. Each picture corresponds to a specific combination of MAFs at the three disease loci as indicated above each plot.

**Figure 8:**
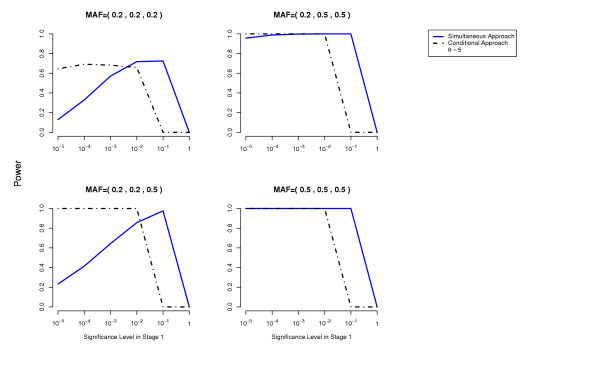
**Three-locus Threshold Model**. Comparison of the conditional approach with the simultaneous approach for the three-locus threshold disease model. *θ *= 5.0. Each picture corresponds to a specific combination of MAFs at the three disease loci as indicated above each plot.

In terms of power, the conditional approach performs generally better (we assume the optimal cutoff has been chosen for each method). This is especially true when the allele frequencies at the disease loci differ, or more generally when the effects at the interacting disease loci are not equal. For example, the threshold disease model when MAF = (0.05,0.5) in Figure 2. Also the missing lethal genotype disease model illustrates very well the situation where one disease locus of mild marginal effect is detected using the conditional approach due to the interaction with another disease locus with stronger marginal effect. If the disease loci have approximately the same importance, the two approaches perform similarly. We have performed power computations also on a three-locus threshold model and report similar results (Figures 7, 8).

### Note

For the conditional approach, for the two-locus disease models that we have simulated, there seems to be no significant loss in power when one uses a less stringent cutoff in Stage 1, in spite of the use of the Bonferroni correction and the increase in the number of tests performed as the threshold in the first stage becomes more liberal. This phenomenon can be explained in part by our conservative correction of the error probability for this type of two-stage (sequential) methods (see Section Methods). In fact for the three-locus threshold model, the power does decrease as the significance cutoff in Stage 1 becomes less stringent (Figures 7, 8). In a real situation, one may want to use more sophisticated methods to control for the Type 1 error (e.g. resampling methods).

Also we mention that in all three disease models that we have simulated, there is some marginal effect at one or all disease loci. If none of the disease loci has any marginal effect, then only the exhaustive approach (all pair-wise interactions) has a chance to detect those. However this approach has real drawbacks: it is computationally intensive and also the multiple-testing problem becomes more serious.

## Conclusion

Complex genetic diseases are believed to be caused by multiple genetic and environmental factors working together to produce susceptibility. The abundance of SNPs (single nucleotide polymorphisms) together with technologies that can measure hundreds of thousands of SNPs across the genome make genome-wide association studies feasible. New statistical methods that can deal with such large-scale studies are needed. Two-stage approaches, where the first stage is used to select promising markers using single-locus methods followed by a second stage where multi-locus methods can be used, are likely to be very useful.

In this report, we presented a simulation study to compare two two-stage approaches: the conditional and the simultaneous approach. Our results suggest that the conditional approach is more robust and performs generally better over a range of disease models when compared with the simultaneous approach. In particular, the conditional approach succeeds in detecting weaker loci that have strong interaction with more obvious loci (that are easy to detect), whereas the simultaneous approach is not powerful in this realistic situation. Hence the two-stage conditional approach is expected to be more powerful than the simultaneous approach when applied to genome-wide association studies.

## Methods

We now present the details of our procedure to compute power for the two approaches: the simultaneous approach and the conditional approach. Both methods are based on the following two-stage algorithm:

### Stage 1

A locus-by-locus search is performed and those markers significant at a certain (marker-wise) level *α *are selected:

*P*(χ22
 MathType@MTEF@5@5@+=feaafiart1ev1aaatCvAUfKttLearuWrP9MDH5MBPbIqV92AaeXatLxBI9gBaebbnrfifHhDYfgasaacH8akY=wiFfYdH8Gipec8Eeeu0xXdbba9frFj0=OqFfea0dXdd9vqai=hGuQ8kuc9pgc9s8qqaq=dirpe0xb9q8qiLsFr0=vr0=vr0dc8meaabaqaciaacaGaaeqabaqabeGadaaakeaaiiGacqWFhpWydaqhaaWcbaGaeGOmaidabaGaeGOmaidaaaaa@307B@ > *k*_*α*_) = *α *(*)

We call this set of markers *S *(for selected) and the entire set of markers *M*.

### Stage 2

For each pair of loci (*l*, *m*) (for the simultaneousapproach both *l *and *m *are from *S*, whereas for the conditional approach *l *∈ *S *and *m *∈ *M*) we fit the full logistic regression model (main effects and interaction).

The power to detect both disease loci with the simultaneous approach at a genome-wide error rate of 0.05 is:

Power_s _= *P*(detect both disease loci at (genome-wide) *α *= 0.05)

= *P*(detect both disease loci in Stage 1 at a certain level)

· *P*(full model significant in Stage 2|both disease loci detected in Stage 1)

Similarly for the conditional approach we have:

Power_c _= *P*(detect both disease loci at (genome-wide) *α = *0.05)

= *P*(detect ≥ 1 disease locus in Stage 1 at a certain level)

· *P*(full model significant in Stage 2| ≥ 1 disease locus detected in Stage 1)

The joint probability, *P*(detect both disease loci in Stage 1 at a certain level), can be estimated by the product of the individual probabilities, i.e.

*P*(detect both disease loci in Stage 1 at a certain level) ≈

≈ *P*(detect locus 1 in Stage 1) · *P*(detect locus 2 in Stage 1).     (1)

This also implies that:

*P*(detect ≥ 1 disease locus in Stage 1 at a certainlevel) ≈

≈ *P*(detect locus 1 in Stage 1) + *P*(detect locus 2 in Stage 1)

- *P*(detect locus 1 in Stage 1) · *P*(detect locus 2 in Stage 1)

### Note

The approximate equal sign in (1) is due to the fact that the data are simulated under epistatic disease models, where the ability to detect a disease locus is dependent not only on the relative risk of that locus, but also on the relative risk of the other disease locus as well. The two-locus interaction multiplicative and threshold models are synerglstic epistatic models where the correlation of the relative risks at the two loci is positive; the missing lethal genotype model is an antagonistic epistatic model, where the correlation is negative. However, the magnitude of the mutual information of the relative risks at the two loci is very small in the simulated disease models.

For Stage 2, when we compute the power to find the full model significant, we use a modified statistic to correct for the fact that the markers in *S *were selected in Stage 1. We make the same correction as in Marchini et al. [2]; namely, if we denote by *R*_(*l*,*m*) _the log-likelihood ratio statistic in Stage 2, we modify it as follows:

Simultaneous approach: R′(l,m)
 MathType@MTEF@5@5@+=feaafiart1ev1aaatCvAUfKttLearuWrP9MDH5MBPbIqV92AaeXatLxBI9gBaebbnrfifHhDYfgasaacH8akY=wiFfYdH8Gipec8Eeeu0xXdbba9frFj0=OqFfea0dXdd9vqai=hGuQ8kuc9pgc9s8qqaq=dirpe0xb9q8qiLsFr0=vr0=vr0dc8meaabaqaciaacaGaaeqabaqabeGadaaakeaacuWGsbGugaqbamaaBaaaleaacqGGOaakcqWGSbaBcqGGSaalcqWGTbqBcqGGPaqkaeqaaaaa@3367@ = *R*_(*l*,*m*) _- 2 · *k*_*α*_

Conditional approach: R′(l,m)
 MathType@MTEF@5@5@+=feaafiart1ev1aaatCvAUfKttLearuWrP9MDH5MBPbIqV92AaeXatLxBI9gBaebbnrfifHhDYfgasaacH8akY=wiFfYdH8Gipec8Eeeu0xXdbba9frFj0=OqFfea0dXdd9vqai=hGuQ8kuc9pgc9s8qqaq=dirpe0xb9q8qiLsFr0=vr0=vr0dc8meaabaqaciaacaGaaeqabaqabeGadaaakeaacuWGsbGugaqbamaaBaaaleaacqGGOaakcqWGSbaBcqGGSaalcqWGTbqBcqGGPaqkaeqaaaaa@3367@ = *R*_(*l*,*m*) _- *k*_*α *_where *k*_*α *_is defined as in (*).

This correction is based on the following facts:

*R*_(*l*,*m*) _≥ *R*_*l *_+ *R*_*m*_

where *R*_*l *_is the log-likelihood ratio statistic for the model with only locus *l*. Also for the conditional approach we only know that *R*_*l *_(*l *∈ *S*) is greater than *k*_*α*_, whereas for the simultaneous approach both *R*_*l *_and *R*_*m *_are greater than *k*_*α*_.

For Stage 2, we use a Bonferroni correction to achieve a genome-wide error rate of at most 0.05. More exactly, for the simultaneous approach the total number of tests in Stage 2 is (αsL2)
 MathType@MTEF@5@5@+=feaafiart1ev1aaatCvAUfKttLearuWrP9MDH5MBPbIqV92AaeXatLxBI9gBaebbnrfifHhDYfgasaacH8akY=wiFfYdH8Gipec8Eeeu0xXdbba9frFj0=OqFfea0dXdd9vqai=hGuQ8kuc9pgc9s8qqaq=dirpe0xb9q8qiLsFr0=vr0=vr0dc8meaabaqaciaacaGaaeqabaqabeGadaaakeaadaqadaqaauaabeqaceaaaeaaiiGacqWFXoqydaWgaaWcbaGaem4CamhabeaakiabdYeambqaaiabikdaYaaaaiaawIcacaGLPaaaaaa@33A0@, where *α*_*s *_is the cutoff used in Stage 1 and *L *is the total number of markers. For the conditional approach the total number of tests in Stage 2 is *α*_*c*_*L*(*L *- *α*_*c*_*L*) + (αcL2)
 MathType@MTEF@5@5@+=feaafiart1ev1aaatCvAUfKttLearuWrP9MDH5MBPbIqV92AaeXatLxBI9gBaebbnrfifHhDYfgasaacH8akY=wiFfYdH8Gipec8Eeeu0xXdbba9frFj0=OqFfea0dXdd9vqai=hGuQ8kuc9pgc9s8qqaq=dirpe0xb9q8qiLsFr0=vr0=vr0dc8meaabaqaciaacaGaaeqabaqabeGadaaakeaadaqadaqaauaabeqaceaaaeaaiiGacqWFXoqydaWgaaWcbaGaem4yamgabeaakiabdYeambqaaiabikdaYaaaaiaawIcacaGLPaaaaaa@3380@, where *α*_*c *_is the cutoff used in Stage 1 of the conditional method.

The approach for the three-locus models is similar.

### Stage 1

A locus-by-locus search is performed and those markers significant at a certain level *α *are selected:

*P*(χ22
 MathType@MTEF@5@5@+=feaafiart1ev1aaatCvAUfKttLearuWrP9MDH5MBPbIqV92AaeXatLxBI9gBaebbnrfifHhDYfgasaacH8akY=wiFfYdH8Gipec8Eeeu0xXdbba9frFj0=OqFfea0dXdd9vqai=hGuQ8kuc9pgc9s8qqaq=dirpe0xb9q8qiLsFr0=vr0=vr0dc8meaabaqaciaacaGaaeqabaqabeGadaaakeaaiiGacqWFhpWydaqhaaWcbaGaeGOmaidabaGaeGOmaidaaaaa@307B@ > *k*_*α*_) = *α*

We call this set of markers *S *(for selected) and the entire set of markers *M*.

### Stage 2

For each triple of loci (*l*, *m*, *n*) (for the simultaneous approach all *l*, *m *and *n *are from *S*, whereas for the conditional approach *l *∈ *S*, *m *∈ *M *and *n *∈ *M*) we fit the full logistic regression model (main effects and interaction).

The power computations are similar to those above, hence we omit the details.

## Authors' contributions

II participated in the design of the study, performed the statistical analysis, and drafted the manuscript. MM conceived the study, and participated in its design and coordination and helped to draft the manuscript. All authors read and approved the final manuscript.
